# Setting policy priorities to address eating disorders and weight stigma: views from the field of eating disorders and the US general public

**DOI:** 10.1186/1471-2458-14-524

**Published:** 2014-05-29

**Authors:** Rebecca M Puhl, Dianne Neumark-Sztainer, S Bryn Austin, Joerg Luedicke, Kelly M King

**Affiliations:** 1 Rudd Center for Food Policy & Obesity, Yale University, 309 Edwards Street, New Haven CT 06520, USA; 2 Division of Epidemiology and Community Health, School of Public Health, University of Minnesota, Minneapolis, USA; 3 Department of Social and Behavioral Sciences, Harvard School of Public Health, and Division of Adolescent and Young Adult Medicine, Boston Children’s Hospital, Boston, USA; 4 Bloomberg School of Public Health, Johns Hopkins University, Baltimore, USA

**Keywords:** Policy, Eating disorders, Prevention, Stigma, Weight, Public support

## Abstract

**Background:**

The prevalence and health consequences of eating disorders and weight stigmatization have prompted increasing discussion of potential policy actions to address these public health issues. The present study aimed to assess support for policy strategies to address eating disorders and weight stigmatization among the general public and relevant health professionals.

**Methods:**

An Internet survey was fielded to a national sample of 944 US adults and 1,420 members of professional organizations specializing in eating disorders to examine their support for 23 potential policy strategies to address eating disorders and weight stigma. Participants also rated policy actions according to their potential for positive impact and feasible implementation.

**Results:**

Support for the majority of health and social policies was high in both samples. For example, strategies to 1) improve school-based health curriculum to include content aimed at preventing eating disorders, 2) require training for educators and health providers on the prevention and early identification of eating disorders, and 3) implement school-based anti-bullying policies that that protect students from being bullied about their weight, were supported by over two-thirds of participants.

**Conclusions:**

Our findings suggest that both health and social policy actions will be important in broader policy initiatives to address eating disorders and weight stigma.

## Background

Eating disorders, such as anorexia nervosa, bulimia nervosa, and binge eating disorder are of serious public health concern given their high prevalence and adverse health consequences [1,2]. Both the acute and chronic psychiatric and medical consequences of eating disorders are well documented and include osteoporosis, cardiovascular, endocrine, gastrointestinal, and skeletal disorders, dental problems, nutritional deficiencies, obesity, psychiatric disorders, and substance use [3,4]. Multiple psychological and biological risk factors can contribute to eating disorders, and socio-cultural factors such as cultural pressures to be thin perpetuated by the media, fashion, and diet industries, and the normalization of dieting and weight-based teasing in modern society, are also significant contributors to eating disorders [5-8].

Adding to the complexity and challenges of the social environment is our culture of stigma related to body weight. Overweight individuals face widespread stigma and discrimination in numerous settings as a result of their weight [9]. Recent estimates indicate that rates of weight discrimination have increased in the United States and are comparable to racial discrimination, especially among women [10,11]. Among adolescents, weight stigma is experienced as victimization and bullying. Reports by educators, parents, and students suggest that weight-based bullying is one of the most common forms of bullying and harassment in the school setting [12-15]. Exposure to weight stigma places overweight children and adults at increased risk for a variety of adverse health consequences, including psychiatric disorders, substance dependence, suicidality, cardiovascular reactivity, unhealthy eating behaviors, obesity, eating disorders, and avoidance of health care [16-22]. Given that experiences of weight stigma and weight-related harassment increase risk for eating disorders [6,19,23] and weight gain [20], these issues are closely linked in important ways, and have direct and damaging consequences for psychological and physical health.

As a result of the growing concern about both eating disorders and weight stigma, and given their high prevalence and public health burden, there have been increasing calls for government policy actions to help reduce and prevent these problems on a broader scale. Experts working in eating disorders or related fields have called for multiple policy interventions and regulations, including efforts to achieve equitable treatment and insurance coverage for eating disorders [24], implement school-based screening for eating disorders [25,26], place government restrictions on access to over-the-counter drugs and supplements for weight control by youths [27], require policies to address eating disorders in college athletic programs [28], curtail weight-related mistreatment and bullying among youth in schools [19,21,23,29,30], and implement legal protections against weight discrimination [31,32]. Fostering political will to trigger government actions in these areas will require increased and strategic advocacy efforts to prioritize eating disorders on policy agendas [33].

Despite these calls for action, policy and legal initiatives related to eating disorders have thus far been limited, with most government actions occurring outside of the U.S. [33,34]. Examples include legal bans on extremely thin fashion models in Israel [35] and Madrid, Spain [36], and codes of conduct established by the state government in Victoria, Australia, to establish media standards for the portrayal of very thin models [37]. Within the U.S., there has been a push by advocacy organizations to introduce the Federal Response to Eliminate Eating Disorders Act (FREED Act) in Congress, which if passed, would provide funding for research, education, and prevention activities and improve access to treatment of eating disorders by requiring treatment coverage to be consistent with coverage of medical benefits [38]. In addition, Virginia passed a law in 2013 requiring schools to promote early detection of eating disorders in youth [39], and California has made it a misdemeanor for coaches to distribute laxatives for the purpose of weight loss to youth athletes [27]. With respect to weight stigma and discrimination, there are no federal laws in the U.S. to prohibit weight discrimination, and Michigan is the only state with such a law in place [40]. State anti-bullying laws and school-based anti-bullying policies vary considerably, with little indication that youth are adequately protected from weight-based bullying. In general, there is little protection or recourse for individuals who have experienced weight discrimination.

Given that policy actions addressing eating disorders and weight stigma have been minimal, but are beginning to emerge, it is important to identify how much support they would receive from the general public and health professionals, as social approval can be a powerful catalyst for the political will necessary to drive policy change [41,42]. Therefore, surveying both the general public and individuals from the eating disorders field is a crucial step to identify which policy actions to prioritize. The present study aimed to assess levels of support for potential policy actions to address eating disorders and weight stigmatization, via an online self-report survey of both individuals from the eating disorders field and the U.S. general public. We also examined participants’ perceptions of the potential impact and feasibility of policy actions, and assessed whether certain socio-demographic characteristics influenced participants’ support for different types of policy actions.

## Methods

### Sample

Study participants were drawn from two distinct samples. First, to assess support for policy actions among the general public, we conducted a survey of a diverse, national sample of U.S. adults, who were recruited through a survey panel administered by Survey Sampling International (SSI; http://www.surveysampling.com). SSI recruits participants through thousands of websites with data aggregators that reach millions of users. Panelists were aged 18 years and older, actively indicated their intention to join an SSI panel, and provided validated geographic and demographic information. SSI set quotas to approximate U.S. Census demographics [43]. Of the 1157 participants who entered the survey, 223 (19%) were excluded due to listwise deletion of item non-response missing data, resulting in a final sample size of 934 participants from the U.S. general public.

Second, to obtain a sample of individuals from the eating disorders field, the survey was advertised on websites, electronic newsletters, and/or list-servs of existing professional organizations in the U.S. that specialize in eating disorders. These organizations included the Academy for Eating Disorders, Binge Eating Disorder Association, and National Eating Disorders Association. Announcements about the study contained a weblink to the online survey. Participants who clicked on the weblink were transferred to the survey website (hosted by Qualtrics.com) and were provided with information explaining the survey and inviting them to participate. Of the 1977 participants who began the survey, 320 (16%) withdrew at the beginning of the survey prior to responding to any questions. Of the remaining 1657 participants, 253 (15%) were excluded due to listwise deletion of item non-response missing data, yielding a final sample of 1404 individuals from the eating disorders field. Table 1 presents sample characteristics.

**Table 1:** Sample characteristics from the general public and eating disorders field (N = 2338)

	**n**	**%/M**
**U.S. General public**		
Female gender	483	51.7
Age (in years)	934	43.3
*Race*		
White	648	69.4
African-American	108	11.6
Hispanic	96	10.3
Other	82	8.8
*Highest level of education*		
High school or less	231	24.7
Some college	340	36.4
College or higher	363	38.9
*Family income*		
Under $25,000	239	25.6
$25,000-$49,999	267	28.6
$50,000-$74,999	188	20.1
$75,000-$99,999	110	11.8
$100,000 or more	130	13.9
*Political affiliation*	260	27.8
Conservative		
Moderate	438	46.9
Liberal	236	25.3
*History of eating disorder*		
Personal history of ED	124	13.3
History of ED in family	153	16.4
Weight status*	31	3.5
Underweight		
Normal weight	309	35.2
Overweight	268	30.5
Obese	270	30.8
Body mass index (kg/m^2^)	878	28.2
**Eating Disorders Field**		
Female gender	1338	95.3
Age (in years)	1404	36.8
*Race*	1282	91.3
White		
African-American	13	0.93
Hispanic	49	3.49
Other	60	4.27
*History of eating disorder*	925	65.9
Personal history of ED		
History of ED in family	675	48.1

Note. ED = eating disorder. Age ranged from 18 to 90 years in the general public sample (SD = 16.5), BMI ranged from 12 to 68 kg/m^2^ (SD = 7.6); among eating disorder experts, age ranged from 18 to 87 years (SD = 4.2).

*Weight status and BMI were categorized using the clinical guidelines for the classification of overweight and obesity in adults by the National Heart, Lung and Blood Institute of the National Institutes of Health, which defines "normal weight" as a BMI (in kg/m^2^) of 18.5–24.9; "overweight" as a BMI of 25.0–29.9; "obese" as a BMI > =30.

All participation was voluntary and anonymous, and participants in both samples completed identical surveys. The survey software (Qualtrics) enabled features to prevent the same user from completing the survey more than once. Data collection occurred during May through July of 2013. All participants provided informed consent, and the study was approved by the Yale University IRB.

### Survey questionnaire

We developed an online self-report survey instrument to assess level of support for potential policy actions to address eating disorders and weight stigmatization. A list of 37 potential policy actions were generated through reviews of the literature, identification of related policies being implemented in other countries, ideas proposed at scientific meetings, and discussions with researchers and advocates in the fields of eating disorders, psychology, and public health. After carefully reviewing this list, items were excluded that were too vague or were redundant with other items (n = 10), resulting in 27 items that formed an initial version of the questionnaire. We fielded this questionnaire to 10 international experts in the eating disorders field, who pre-tested survey questions and provided feedback on wording and content for each item. Based on their feedback, item wording was revised for increased clarity and four items were removed, yielding a total of 23 questions. The survey asked participants to indicate the extent of their support (on a 5-point Likert rating scale, ranging from 1 = definitely oppose to 5 = definitely support) for each of 23 potential policy actions related to eating disorders and weight stigmatization. Scale items were later recoded into binary items to assess the percentage of participants who either "somewhat" or "definitely" supported each policy action (reflecting a "4" or "5" on the 5-point Likert rating scale).

Policy actions were focused in five different content areas (see Table 2): 1) schools (e.g., "*Schools should conduct screening for eating disorders*"), 2) weight stigma and discrimination (e.g., "*Existing civil rights laws should include body weight to protect people from weight discrimination*"), 3) healthcare (e.g., "*Insurance companies should be required to reimburse for eating disorder treatment*"), 4) weight control products (e.g., "*Selling over-the-counter diet pills and laxatives to minors should be restricted by the government*"), and 5) the media (e.g., "*The use of very underweight fashion models should be restricted by the government*").

**Table 2:** Support for policy actions among participants from the general public and the eating disorders field

		Percent (%) of participants who support policy*	
**Item number**	**Policy actions**	**Eating disorders field**	**General public**
	**School-based initiatives**		
1	Schools should conduct screening for eating disorders.	76.7	52.5
2	Schools should have anti-bullying policies that protect students from being bullied about their weight.	96.1	83.4
3	School-based health curriculum should include content aimed at preventing eating disorders.	95.3	77.3
4	Schools should measure students’ height/weight for the purpose of reporting to families their child's weight status.	22.1	44.2
5	Schools should measure students’ height and weight to monitor population changes over time.	21.6	29.7
6	School sports coaches should receive training about the prevention and early identification of eating disorders.	98.5	70.8
	**Weight stigma and discrimination**		
7	Existing civil rights laws should include body weight to protect people from weight discrimination.	74.3	50.7
8	It should be illegal for an employer to refuse to hire a qualified person because of his/her body weight.	84.9	69.2
9	The government should have laws in place to protect people from weight discrimination in the workplace.	88.8	70.6
10	Existing anti-bullying laws should be modified to include protections for youth who are bullied about their weight.	94.6	76.8
11	Campaigns or interventions that address obesity should avoid content that stigmatizes overweight people.	89.4	61.5
	**Healthcare**		
12	Insurance companies should be required to reimburse for eating disorder treatment.	98.6	51.5
13	Insurance companies should be required to reimburse for obesity treatment.	84.5	52.4
14	Restrictions should be placed on elective, cosmetic surgery for minors, except when medically recommended.	84.7	70.5
15	Healthcare providers should be trained on the prevention and early identification of eating disorders.	99.6	79.8
16	Dentists should be trained to screen for signs and symptoms of eating disorders.	95.6	64.6
17	Health care providers should receive sensitivity training to prevent weight stigma in their clinical practice.	97.8	67
	**Weight Control Products**		
18	Weight loss claims about diet products and weight loss programs should be regulated by the government.	81.7	58
19	Selling over-the-counter diet pills and laxatives to minors should be restricted by the government.	85.6	64.9
20	Selling muscle enhancers to minors (e.g., creatine, protein powders) should be restricted by the government.	75.8	63.9
	**Media**		
21	Magazines targeting readers under 18 years of age should be prohibited from advertising weight loss products.	89.8	51.9
22	The media should be required to include disclaimers for photographs of models that have been digitally altered.	91.4	67.3
23	The use of very underweight fashion models should be restricted by the government.	71.1	42.7

*Percentages indicate participants who "Somewhat Supported" or "Definitely Supported" each policy action.

After indicating their level of support for each policy action, participants were asked to choose the five policy actions from the list of all 23 policies that they believed would have the most positive impact on efforts to address weight stigma and eating disorders. Participants were then asked to select the five policy actions from the full list of 23 policies that they believed would be the most feasible to implement.

Participants also responded to questions assessing demographic characteristics. Participants in the general public sample were asked to indicate their age, gender, race/ethnicity, height and weight, level of education, household income, and political orientation. Participants recruited from the eating disorders field were asked their gender, age, ethnicity, and their profession. Both samples were additionally asked whether they, or anyone in their family, have had an eating disorder.

### Analysis

Descriptive statistics and regression models to assess demographic predictors of policy support were used for analyzing the data. Since the study outcome variables (mean scale scores derived by averaging across respective items within each of the five content areas described above) were negatively skewed with high probability mass around their theoretical maximum score, tobit models for censored data were used [44]. Separate models were fit for each of the five outcome variables and for each of the two samples. For the general public sample, missing values for BMI were multiply imputed (20 datasets) [45], utilizing information from all outcome and predictor variables that were used in the regression analyses (Table 3). All analyses were performed using the statistical analysis software Stata, version 13.

**Table 3:** Support for policy actions across five policy content areas among participants from the U.S

	**School-based initiatives**	**Weight stigma**	**Health-care**	**Weight control products**	**Media**
***U.S. General Public***					
Gender (ref. male)	.	.	.	.	.
Female	0.121*	0.308***	0.257***	0.218*	0.472***
Personal history of ED	.	.	.	.	.
Yes	0.057	0.187	0.217*	0.017	0.061
History of ED in family	.	.	.	.	.
Yes	0.114	0.082	0.033	0.053	0.143
Race/Ethnicity (ref. White)	.	.	.	.	.
African-American	0.256**	0.275*	-0.002	0.206	-0.031
Hispanic	-0.084	-0.078	-0.198*	-0.052	-0.120
Other	0.011	-0.009	-0.160	-0.006	-0.029
Highest educational degree (ref. High school or less)	.	.	.	.	.
Some college	-0.002	-0.089	0.190*	0.115	-0.034
College or higher	0.106	-0.083	0.287***	-0.010	-0.021
Current household income (ref. <$25,000)	.	.	.	.	.
$25,000-$49,999	0.134	0.145	0.065	0.193	0.225*
$50,000-$74,999	0.006	0.014	-0.018	0.379**	0.045
$75,000-$99,999	0.080	0.166	0.135	0.130	0.107
100,000 or more	0.118	0.078	0.060	0.273	0.085
Political orientation (ref. conservative)	.	.	.	.	.
Moderate	0.141*	0.248**	0.067	0.312**	0.216*
Liberal	0.204*	0.440***	0.272***	0.313**	0.275**
Age (in years)	0.000	-0.002	0.001	0.007**	-0.001
BMI (kg/m^2^)	0.004	0.030***	0.013**	0.016**	0.015**
Constant	3.587***	2.733***	3.057***	2.548***	2.723***
σ	0.881***	1.067***	0.865***	1.232***	1.106***
N	934	934	934	934	934
***Participants from Eating Disorders Field***					
Gender (ref. male)	.	.	.	.	.
Female	0.184*	0.216	0.119*	0.399*	0.701***
Personal history of ED	.	.	.	.	.
Yes	0.028	0.166**	0.005	-0.075	-0.011
History of ED in family	.	.	.	.	.
Yes	0.136***	-0.022	0.041	0.028	0.151*
Age (in years)	0.000	-0.001	0.006***	0.012***	-0.003
Race (ref. other)	.	.	.	.	.
White	0.039	-0.070	-0.078	-0.107	-0.010
Constant	4.449***	4.484***	4.537***	3.883***	4.146***
σ	0.604***	0.913***	0.423***	1.233***	1.096***
N	1404	1404	1404	1404	1404

*Note*. Shown are raw coefficients from tobit models; outcome variables are censored from above; ref. = reference category. For the sample of participants from the eating disorders field, race/ethnicity was dichotomized into Whites vs. "other" due to low prevalence of non-Whites. Significance levels: *p < 0.05, **p < 0.01, ***p < 0.001.

## Results

### Sample characteristics

Table 1 presents a summary of sample characteristics. The general public sample approximated 2010 U.S. Census demographics in terms of gender, race, and household income [46]. Additionally, weight status categories approximate the U.S. adult population [47]. Participants from the eating disorders field characterized their professions as psychologists (9%), social workers (9%), patient advocates (9%), dietitians (8%), researchers (4%), physicians (1%), psychiatrists (1%), and students (24%). The remaining 34% of the sample classified their health profession in an "other" category, and self-described their profession to be one of the following: health educators, nurses, professional counselors, mental health therapists, fitness professionals, professors, public health professionals, and health coaches.

### Support for policy actions

Table 2 presents the percentages of participants in each sample who indicated support for the 23 policy actions. Percentages reflect those participants who "somewhat" or "definitely" supported each policy action (reflecting a "4" or "5" on the 5-point Likert rating scale). Overall, both samples expressed considerable support for most policy actions. Among the general public, the majority of participants (50%-83%) expressed support for 20 of the 23 policy actions, with the most support for anti-bullying policies that protect youth from weight-based bullying and policies to implement prevention and training for the early identification of eating disorders among health care providers, schools, and sports coaches. Policies generating the least public support (29-44%) included policies for government restriction of underweight fashion models and those requiring schools to measure and report students’ body weight.

Among participants from the eating disorders field, 71%-99% expressed support for 21 of the 23 policy actions. Participants in this sample were highly supportive of policy actions to address eating disorders in schools, healthcare settings, and the media, as well as policy and legal measures to address weight-based bullying and discrimination. As with the general public, policies generating the least support (22% of participants) were those requiring schools to measure and report students’ body weight.

### Perceived impact of policy actions

**Figure 1:**
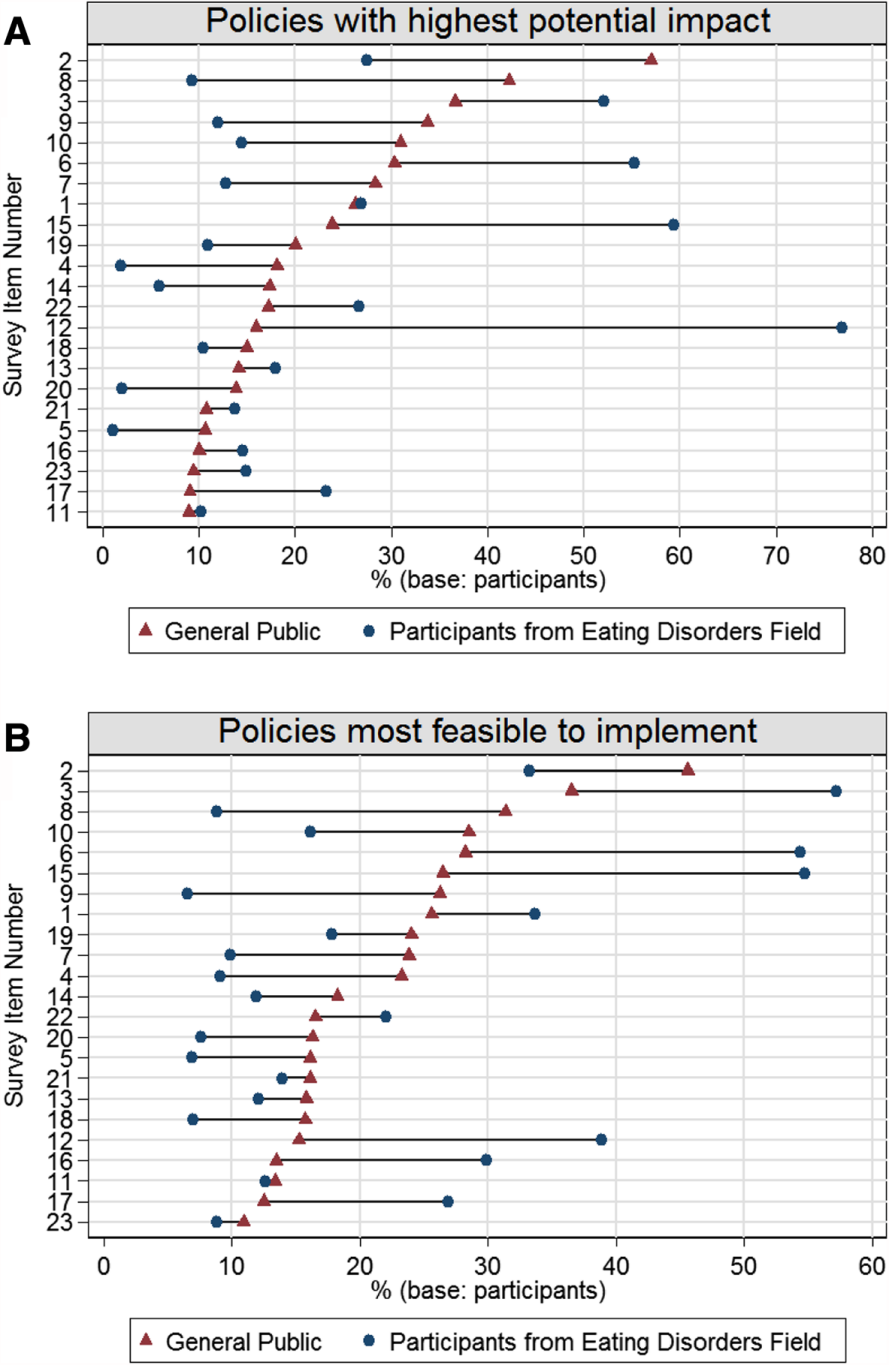
**Perceptions of potential impact and feasibility of policy actions among participants from the U.S. General Public and the Eating Disorders Field (Corresponding content for survey item numbers is presented in Table **2**).**

Figure 1 presents the percentage of participants in each sample who selected each policy among the top 5 policy actions to 1) be most likely to have the highest impact and 2) be most feasible to implement. Among the general public, the top 5 policy actions selected to be most likely to have the highest impact included the following: 1) "*Schools should have anti-bullying policies to protect students from weight-based bullying*" (selected by 57% of participants), 2) "*It should be illegal for an employer to refuse to hire a qualified person because of his/her body weight"* (42%), 3) "*School-based health curriculum should include content to address eating disorders"* (37%), 4) "*The government should have laws in place to protect individuals from weight discrimination in the workplace"* (34%), and 5) "*Existing anti-bullying laws should be modified to include protections for youth who are bullied about their weight"* (31%).

Among participants from the eating disorders field, the top 5 policy actions selected for highest potential impact were as follows: 1) "*Insurance companies should be required to reimburse for treatment of eating disorders"* (77%), 2) "*Healthcare providers should be trained about the prevention and early identification of eating disorders"* (59%), 3) *"School sports coaches should be trained about the prevention and early identification of eating disorders*"(52%), 4) *"School-based health curriculum should include content to address eating disorders"* (52%), and 5) *"Schools should have anti-bullying policies to protect students from weight-based bullying"* (27%).

### Perceived feasibility of policy actions

Regarding policies perceived to be most feasible to implement, very similar findings emerged. Four of the same 5 policies that each sample rated most highly for potential impact were also selected as being the most feasible to implement. Among the general public, policies that were selected to be most feasible to implement included: 1) "*Schools should have anti-bullying policies to protect students from weight-based bullying"* (46%), 2) "*School-based health curriculum should include content to address eating disorders"* (37%), 3) "*It should be illegal for an employer to refuse to hire a qualified person because of his/her body weight"* (31%), 4) *"Existing anti-bullying laws should be modified to include protections for youth who are bullied about their weight"* (29%), and 5) *"School sports coaches should receive training about the prevention and early identification of eating disorders"* (28%). Among participants from the eating disorders field, policies selected to be most feasible to implement were: 1) *"School-based health curriculum should include content to address eating disorders"* (57%), 2) *"Healthcare providers should be trained about the prevention and early identification of eating disorders"* (55%), 3) *"School sports coaches should receive training about the prevention and early identification of eating disorders"* (54%), 4) *"Insurance companies should be required to reimburse for treatment of eating disorders"* (39%), and 5) *"Schools should conduct screening for eating disorders"* (34%).

### Regression analyses

Table 3 presents results from five separate tobit regression models fit to the data for the survey responses among the general public and, separately, participants from the eating disorders field, across the five policy content areas. The two policies addressing BMI reporting in schools were excluded from this analysis due to the little support they received from both samples. Among the general public, regression results showed that women expressed greater support than men across all policy content areas. African-Americans expressed higher support than Whites on policies addressing both schools and weight stigma, while Hispanic participants expressed less support than Whites on healthcare-related policies. The weight status of participants was positively associated with higher support on all policy dimensions, except for school-related policies. Participants with higher educational attainment expressed more support for healthcare- related policies compared to participants with a high school degree or less. Participants with a personal eating disorder history also expressed more support for healthcare-related policies than those without this history. Finally, participants who classified themselves as having a moderate or liberal political orientation also expressed more support across policy dimensions compared to conservatives.

Similar findings emerged among participants from the eating disorders field. Women expressed higher policy support than men across all five policy content areas (although the gender effect on policies addressing weight stigma was not statistically significant). Participants with a personal history of an eating disorder expressed slightly higher support of policies addressing weight stigma than those without this history. In addition, participants who indicated that someone in their family had experienced an eating disorder were more likely than those without this family history to support school- and media-related policies. Finally, support for policies to improve quality of and access to healthcare and policies that would place restrictions on youths’ access to weight control products increased with participants' age.

## Discussion

In recent years there has been increased interest and efforts in the use of policy to address obesity; however, similar steps have been slower to emerge to address eating disorders and weight stigma. This study provides needed data on general public and expert support for potential policy actions to guide such efforts. To our knowledge, our study is the first to systematically assess support for policy measures related to eating disorders and to compare policy support among the general public and individuals from relevant health fields. Overall, our findings indicate considerable support in both the eating disorders field and the general public for policy actions to address eating disorders and weight stigmatization across a range of domains, including schools, healthcare, the media, and discrimination laws. Of particular note, strong majorities of individuals from the eating disorders field (ranging from 71-99%) expressed support for 21 of the 23 policy actions that were considered. These findings suggest that the field is supportive of utilizing policy to achieve meaningful change. The considerable support among the general public for most policies (50-83%) is also indicative of the substantial concerns about eating disorders and weight stigma, and provides a platform for moving forward with policy implementation and evaluation. Taken together, these findings may reflect consensus about the socio-cultural forces in our environment that contribute to eating disorders and weight stigmatization and the need for policy measures to address aspects of our environment that contribute to these problems.

Policies requiring 1) school-based health curriculum to include content aimed at preventing eating disorders, 2) training for sports coaches on the prevention of eating disorders, and 3) implementation of school-based anti-bullying policies that protect students from being bullied about their weight were selected as having high potential impact *and* feasibility by both the general public and individuals from the eating disorders field. This suggests that policy measures aiming to address and prevent these problems among youth in the school setting should be prioritized. With increasing national attention to both youth bullying [48] and to improving standards for nutrition and wellness in schools [49], there may be realistic and timely opportunities to implement such policies into existing state and district-level, school-based wellness policies and/or anti-bullying policies.

Given recent policies requiring school-based measurement of students’ heights and weights for the assessment of overweight or obese status, and the potential concerns about this practice for eating disorders and weight stigma, we assessed level of support for these initiatives. Our findings show considerable lack of support (in both samples) for policies to measure children’s height and weight in schools for the purposes of reporting to families their child’s weight status or for aggregating this data and monitoring population changes over time. There has been fervent debate about BMI reporting in schools [50], with concerns that measuring children’s weights in school and sending them home with "BMI report cards" will promote stigma and bullying [51]. As an example, Massachusetts recently halted state efforts by schools to inform parents of their child’s BMI because of bullying concerns [52], although the state continues to support height and weight measurement of students as an important tool for population health monitoring. Our findings suggest that continuing policy initiatives to promote BMI reporting to parents of schoolchildren may be met with opposition.

Finally, it is noteworthy that women expressed higher policy support than men across all five policy content areas. Although previous research has demonstrated similar gender differences in sociopolitical attitudes more generally [53], in the present study it may be that women were more supportive of policies given that they are more likely to experience body image concerns, eating disorders, and weight stigmatization compared to men [10,54]. In light of these observed gender differences, it will be important for advocacy efforts to appeal to both men and women in policy decision-making roles, to provide them with education on the seriousness of both eating disorders and weight stigmatization, and to demonstrate the high level of public support for multiple policy initiatives aimed at their prevention and reduction.

### Directions for future research and policy

Our findings clearly revealed that substantial support already exists for many potential initiatives to address eating disorders and weight stigmatization, indicating that one of the critical conditions needed for generating political will for policy change is well-established. Next steps for scientists and health professionals will entail establishing other important conditions to build political will, including documenting feasibility and effectiveness of viable policy initiatives, examining financial implications of different initiatives, and legal avenues for enacting change through law and/or regulation [41,42]. Rigorous policy-related research and advocacy by health professionals and the general public are key for catalyzing action by policymakers.

## Conclusions

The findings in this study indicate that there is substantial support in both the eating disorders field and the general public for policy actions to address eating disorders and weight stigmatization across multiple areas, including schools, healthcare, the media, and discrimination laws. In particular, policies requiring school-based health curriculum on preventing eating disorders, training for sports coaches on the prevention of eating disorders, and implementation of school-based anti-bullying policies to address weight-based bullying were selected as having high potential impact *and* feasibility by both the general public and individuals from the eating disorders field. These findings offer new insights to guide policy priorities, suggesting that to address eating disorders and weight stigma, both health and social policy actions will be important to include in future policy innovations.

## Competing interests

The authors have no competing interests to disclose.

## Authors’ contributions

RP and DN-S conceptualized the study. RP, DN-S, and SBA designed the survey instrument and prepared the manuscript. JL prepared the dataset, carried out statistical analysis and interpretation, and contributed to manuscript preparation. KK contributed to survey instrument development and oversaw data collection. All authors read and approved the manuscript.

## Pre-publication history

The pre-publication history for this paper can be accessed here:

http://www.biomedcentral.com/1471-2458/14/524/prepub

## References

[B1] Arcelus J Mitchell AJ Wales J Nielsen S Mortality rates in patients with Anorexia Nervosa and other eating disorders: a meta-analysis of 36 studies Arch Gen Psychiatry 2011 68 724 731 10.1001/archgenpsychiatry.2011.74 21727255

[B2] Croll J Neumark-Sztainer D Story M Ireland M Prevalence and risk and protective factors related to disordered eating behaviors among adolescents: relationship to gender and ethnicity J Adolesc Health 2002 31 166 175 10.1016/S1054-139X(02)00368-3 12127387

[B3] Hudson JI Hiripi E Pope HG Kessler RC The prevalence and correlates of eating disorders in the national comorbidity survey replication Biol Psychiatry 2007 61 348 358 10.1016/j.biopsych.2006.03.040 16815322 PMC1892232

[B4] Treasure J Claudino AM Zucker N Eating disorders Lancet 2010 375 583 593 10.1016/S0140-6736(09)61748-7 19931176

[B5] Grabe S Hyde JS Ward LM The role of the media in body image concerns among women: a meta-analysis of experimental and correlational studies Psychol Bull 2008 134 460 476 18444705 10.1037/0033-2909.134.3.460

[B6] Haines J Kleinman KP Rifas-Shiman SL Field AE Austin SB Examination of shared risk and protective factors for overweight and disordered eating among adolescents Arch Pediat Adol Med 2010 164 336 343 10.1001/archpediatrics.2010.19 PMC3093706 20368486

[B7] Hesse-Biber S Leavy P Quinn CE Zoino J The mass marketing of disordered eating and eating disorders: the social psychology of women, thinness, and culture Women’s Stud Int Forum 2006 29 208 224 10.1016/j.wsif.2006.03.007

[B8] Keel PK Forney KJ Psychosocial risk factors for eating disorders Int J Eat Disord 2013 46 433 439 10.1002/eat.22094 23658086

[B9] Puhl RM Heuer CA The stigma of obesity: a review and update Obesity 2009 7 941 964 19165161 10.1038/oby.2008.636

[B10] Puhl RM Andreyeva T Brownell KD Perceptions of weight discrimination: prevalence and comparison to race and gender discrimination in America Int J Obesity 2008 32 992 1001 10.1038/ijo.2008.22 18317471

[B11] Andreyeva T Puhl RM Brownell KD Changes in perceived weight discrimination among Americans, 1995–1996 through 2004–2006 Obesity 2008 16 1129 1134 10.1038/oby.2008.35 18356847

[B12] Puhl RM Luedicke J Heuer C Weight-based victimization toward overweight and obese adolescents: observations and reactions of peers J School Health 2011 81 696 703 10.1111/j.1746-1561.2011.00646.x 21972990

[B13] Bradshaw CP Waasdorp TE O’Brennan LM Gulemetova M Findings from the national education association’s nationwide study of bullying: teachers’ and education support professionals’ perspectives National Education Association 2011 http://www.nea.org/assets/docs/Nationwide_Bullying_Research_Findings.pdf PMC4235229 25414539

[B14] Puhl RM Luedicke J Parental opinions and concerns about weight-based bullying in youth Childhood Obesity 2013 9 1 9 doi:10.1089/chi.2013.006423373874

[B15] Bucchianeri MM Eisenberg ME Neumark-Sztainer D Weightism, racism, classism, and sexism: Shared forms of harassment in adolescents J Adolesc Health 2013 5 47 53 23566562 10.1016/j.jadohealth.2013.01.006 PMC3691304

[B16] Bucchianeri MM Eisenberg ME Wall MM Piran N Neumark-Sztainer D Multiple types of harassment: associations with emotional well-being and unhealthy behaviors in adolescents J Adolesc Healthin press 10.1016/j.jadohealth.2013.10.205 PMC4107652 24411820

[B17] Amy NK Aalborg A Lyons P Keranen L Barriers to routine gynecological cancer screening for white and African-American obese women Int J Obes 2006 30 147 155 10.1038/sj.ijo.0803105 16231037

[B18] Hatzenbuehler ML Keyes KM Hasin DS Associations between perceived weight discrimination and the prevalence of psychiatric disorders in the general population Obesity 2009 17 2033 2039 10.1038/oby.2009.131 19390520 PMC3767420

[B19] Neumark-Sztainer D Falkner N Story M Perry C Hannan PJ Mulert S Weight-teasing among adolescents: correlations with weight status and disordered eating behaviors Int J Obesity 2002 26 123 131 10.1038/sj.ijo.0801853 11791157

[B20] Sutin AR Terracciano A Percevied weight discrimination and obesity PLoS One 2013 8 e70048 doi:10.1371/journal.pone.0070048 10.1371/journal.pone.0070048 23894586 PMC3722198

[B21] Quick VM McWilliams R Byrd-Bredbenner C Fatty, fatty, two-by-four: weight-teasing history and disturbed eating in young adult women Am J Public Health 2013 103 508 515 10.2105/AJPH.2012.300898 23327257 PMC3673498

[B22] Puhl R Latner J Obesity, stigma, and the health of the nation’s children Psychol Bull 2007 133 557 580 17592956 10.1037/0033-2909.133.4.557

[B23] Haines J Neumark-Sztainer D Eisenberg ME Hannan PJ Weight teasing and disordered eating behaviors in adolescents: longitudinal findings from project EAT (Eating Among Teens) Pediatrics 2005 117 e209 e215 16452330 10.1542/peds.2005-1242

[B24] Marques L Alegria M Becker AE Chen C Fang A Chosak A Diniz JB Comparative prevalence, correlates of impairment, and service utilization for eating disorders across US ethnic groups: implications for reducing ethnic disparities in health care access for eating disorders Int J Eat Disord 2011 44 412 420 10.1002/eat.20787 20665700 PMC3011052

[B25] Austin SB Ziyaden NJ Forman S Prokop LA Keliher A Jacobs D Screening high school students for eating disorders: result of a national initiative Prev Chronic Dis 2008 5 1 10 PMC2578782 18793502

[B26] Haines J Ziyadeh NJ Franko DL McDonald J Mond JM Austin SB Screening high school students for eating disorders: validity of brief behavioral and attitudinal measures J School Health 2011 81 530 535 10.1111/j.1746-1561.2011.00623.x 21831065

[B27] Pomeranz JL Taylor LM Austin SB Over-the-counter and out-of-control: legal strategies to protect youths from abusing products for weight control Am J Public Health 2013 103 220 225 10.2105/AJPH.2012.300962 23237149 PMC3558759

[B28] Vaughan JL King KA Cottrell RR Collegiate athletic trainers’ confidence in helping female athletes with eating disorders J Athletic Training 2004 39 71 76 PMC385264 15085214

[B29] Eisenberg M Neumark-Sztainer D Peer harassment and disordered eating Int J Adolesc Med Health 2008 20 155 164 18714553 10.1515/ijamh.2008.20.2.155

[B30] Li W Rukavina PB The nature, occurring contexts, and psychological implications of weight-related teasing in urban physical education programs Res Q Exerc Sport 2012 83 308 317 10.1080/02701367.2012.10599862 22808717

[B31] Roehling MV Roehling PV Pichler S The relationship between body weight and perceived weight-related employment discrimination: the role of sex and race J Vocational Behavior 2007 71 300 318 10.1016/j.jvb.2007.04.008

[B32] Puhl RM Heuer C Public opinion about laws to prohibit weight discrimination in the United States Obesity 2011 19 74 82 10.1038/oby.2010.126 20508626

[B33] Austin SB A public health approach to eating disorders prevention: it’s time for public health professionals to take a seat at the table BMC Public Health 2012 12 854 10.1186/1471-2458-12-854 23043459 PMC3519713

[B34] Sánchez-Carracedo D Neumark-Sztainer D López-Guimerà G Integrated prevention of obesity and eating disorders: barriers, developments and opportunities Public Health Nutr 2012 15 2295 10.1017/S1368980012000705 22455792 PMC10271554

[B35] Bruno N Israeli law bans skinny, BMI-challenged models ABC News 2013 http://abcnews.go.com/International/israeli-law-bans-skinny-bmi-challenged-models/story?id=18116291

[B36] Anonymous Madrid bans waifs from catwalks 2006 http://news.bbc.co.uk/2/hi/5341202.stm

[B37] Paxton SJ Cash T, Smolak L Public policy and prevention Body image: a handbook of science, practice and prevention 2011 2 New York: Guilford Press 460 468

[B38] Eating Disorders Coalition FREED Act Introduced 2013 http://www.eatingdisorderscoalition.org/documents/FreedAct.pdf

[B39] Anonymous Schools address important information about eating disorders Richmond Daily-Monitor 2013 http://www.richmond.daily-monitor.com/schools-address-important-information-about-eating-disorders/9050

[B40] Pomeranz JL Puhl RM New developments in the law for obesity discrimination protection Obesity 2013 21 469 471 10.1002/oby.20094 23592654

[B41] Mello MM Wood J Burris S Wagenaar AC Ibrahim JK Swanson JW Critical opportunities for public health law: a call for action Am J Pub Health 2013 103 1979 1988 10.2105/AJPH.2013.301281 24028265 PMC3828690

[B42] Mello MM Studdert DM Brennan TA Obesity – the new frontier of public health law New Engl J Med 2006 354 2601 2610 10.1056/NEJMhpr060227 16775242

[B43] Survey Sampling International The science of samplingc2011 http://www.surveysampling.com/

[B44] McDonald JF Moffitt RA The uses of tobit analysis Rev Econ Stat 1982 62 318 321

[B45] Rubin DB Multiple imputation after 18+ years J Am Statistical Assoc 1996 91 473 489 10.1080/01621459.1996.10476908

[B46] Bureau USC Profile of general population and housing characteristicshttp://www.census.gov/prod/www/decennial.html

[B47] Flegal KM Carroll MD Kit BK Ogden CL Prevalence of obesity and trends in the distribution of body mass index among US adults, 1999–2010 JAMA 2012 307 E1 E7 10.1001/jama.2012.39 22253363

[B48] Shepherd S White House conference tackles bullying CNN Politics 2011 http://www.cnn.com/2011/POLITICS/03/10/obama.bullying/

[B49] Story M Nanney MS Schwartz MB Schools and obesity prevention: creating school environments and policies to promote healthy eating and physical activity Milbank Q 2009 87 71 100 10.1111/j.1468-0009.2009.00548.x 19298416 PMC2879179

[B50] Nihiser AJ Lee SM Wechsler H McKenna M Odom E Reinold C Thomson D Grummer-Strawn L Body mass index measurement in schools J School Health 2007 77 651 671 10.1111/j.1746-1561.2007.00249.x 18076411

[B51] Bidwell A Report: ‘Fat letters’ necessary to fight childhood obesity 2013 http://www.usnews.com/news/articles/2013/08/19/report-fat-letters-necessary-to-fight-childhood-obesity

[B52] Bushak L ‘Fat letters’ to be eliminated in Massachusetts: Public schools will no longer tell parents whether their kids are obese 2013 http://www.medicaldaily.com/fat-letters-be-eliminated-massachusetts-public-schools-will-no-longer-tell-parents-whether-their

[B53] Eagly AH Diekman AB Johannesen-Schmidt MC Koenig AM Gender gaps in sociopolitical attitudes: a social psychological analysis J Pers Soc Psychol 2004 87 796 816 15598107 10.1037/0022-3514.87.6.796

[B54] Neumark-Sztainer D Hannan PJ Weight-related behaviors among adolescent girls and boys: results from a national survey Arch Pediatr Adolesc Med 2000 154 569 577 10.1001/archpedi.154.6.569 10850503

